# Variation in Mating Dynamics across Five Species of Leiobunine Harvestmen (Arachnida: Opliones)

**DOI:** 10.3390/biology7020036

**Published:** 2018-06-14

**Authors:** Kasey D. Fowler-Finn, Sarah L. Boyer, Raine Ikagawa, Timothy Jeffries, Penelope C. Kahn, Eva M. Larsen, Daniel Lee, Morgan Smeester

**Affiliations:** 1 Department of Biology, Saint Louis University, Saint Louis, MO 63103, USA; tijeffr@siue.edu (T.J.); dlee48@slu.edu (D.L.); morgan.smeester@unmc.edu (M.S.); 2 Biology Department, Macalester College, Saint Paul, MN 55105, USA; boyer@macalester.edu (S.L.B.); raineikagawa@gmail.com (R.I.); pcbkahn@gmail.com (P.C.K.); evamarie14@gmail.com (E.M.L.)

**Keywords:** mate choice evolution, mutual assessment, male–female antagonism, Opiliones

## Abstract

The study of mating choices often focuses on correlates of traits to the overall outcome of a mating interaction. However, mating interactions can proceed through a series of stages, with opportunities for assessment at each stage. We compared whether male or female size predicted mating interaction outcome across several stages of mating in five species of North American leiobunine harvestmen (commonly known as daddy longlegs). Leiobunine harvestmen have been previously shown to exhibit incredible morphological diversity consistent with a spectrum of male–female antagonism. Across all of the species, we found a general progression of female size predicting the outcome (success and timing) of early stages of interactions, and male size or male size relative to female size predicting the outcome and timing of later stages of interactions. We also found that size was not a strong predictor of outcome in the two species on the lower end of the antagonism spectrum. The variation in how female and male size predicted outcomes across species and stages of mating suggests that multiple mechanisms may operate to shape mating dynamics within and across species. Given the close relatedness of the species studied, the patterns we uncovered suggest a rapid evolution of the traits and processes predicting the outcome of mating interactions.

## 1. Introduction

The extravagance and rapid evolution of sexual traits have captured the interest of biologists dating back to Darwin [1]. Behavioral and morphological traits used to coordinate sex are often the most divergent aspects of animal phenotype across populations and species [2,3,4,5,6,7,8]. One factor contributing to this extreme diversity is the range of sources of selection that can shape reproductive traits, including precopulatory choice [9], conflict over mating [10,11], pericopulatory choice, postcopulatory choice [2,12], and sperm competition [13,14]. These various sources of selection can differ across species, but they can also operate simultaneously within a single species [15].

Also contributing to the diversity of behaviors involved in mating is the exchange of information between males and females as they assess each other [4,16,17,18]. These male-female interactions can occur over a series of mating stages—roughly broken into precopulatory, copulatory, and postcopulatory—with females and/or males assessing different traits at each stage to determine the amount of time or resources that they will continue to invest in the interaction. As a result, the sources of selection shaping sexual traits can vary across the stages of mating interactions and shape different traits [9,18,19,20]. Across different stages of mating, the selection of behavioral and morphological traits can reinforce each other [21], or act antagonistically [22], further contributing to patterns of diversity across species.

Mating dynamics in the leiobunine harvestmen of North America (commonly known as daddy longlegs) provide an excellent system to study the evolution of mate choice and selection on multiple sexual traits for several reasons. First, genitalic and male armament diversity (i.e., male pedipalps used to hook the female in early stages of mating interactions) suggest variation in mating behavior [23,24,25], specifically along a continuum of mating antagonism, from low in some species to high in others [24]. In species on the high end of the antagonism spectrum, the male penes are capable of applying greater biomechanical force, being longer and stiffer than those from the low end of the spectrum; females have sclerotized pregenital barriers that are capable of blocking forced mating attempts; and, the pedipalps are more sexually dimorphic, with males having larger pedipalps than females [23,24]. On the lower end of the antagonism spectrum, male penes are more flexible and females lack pregenital barriers [23,24]. In all of the species in the clade, males deliver nuptial gifts to the female oral opening prior to mating; however, on the lower end of the antagonism spectrum, male penes have specialized sacs that allow for the easy delivery of a nuptial gift to the female oral opening prior to intromission, whereas these structures have been secondarily lost in non-sacculate species on the higher end of the antagonism spectrum [23,24].

**Figure 1:**
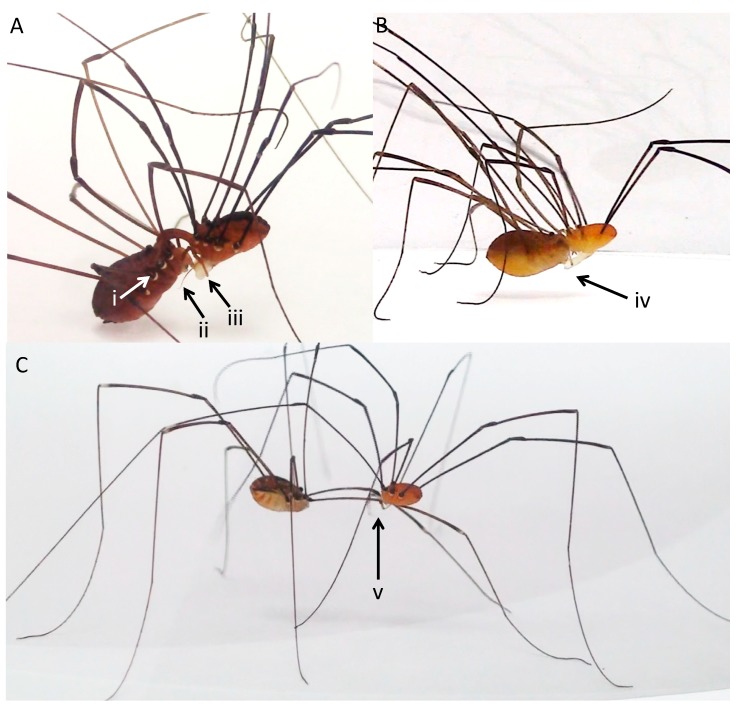
Pairs of leiobunine harvestmen in various stages of mating, with males on the right for all of the pairs pictured. (**A**) A male *L. vittatum* prior to intromission, but after the mating embrace is achieved. Pictured is (i) the male pedipalps hooked behind the coxae of the female’s second legs, (ii) the male’s penis everted prior to intromission, and (iii) the female grabbing the structures on the male hematadocha with her pedipalps. (**B**) The female *L. politum* is touching the male’s fully inflated hematadocha during intromission (iv). Male *L. politum* lack the specialized structures on the hematadocha possessed by male *L. vittatum*. (**C**) Male *L. aldrichi* guarding the female after copulation by grabbing her second leg in his chelicerae (v).

**Figure 2:**
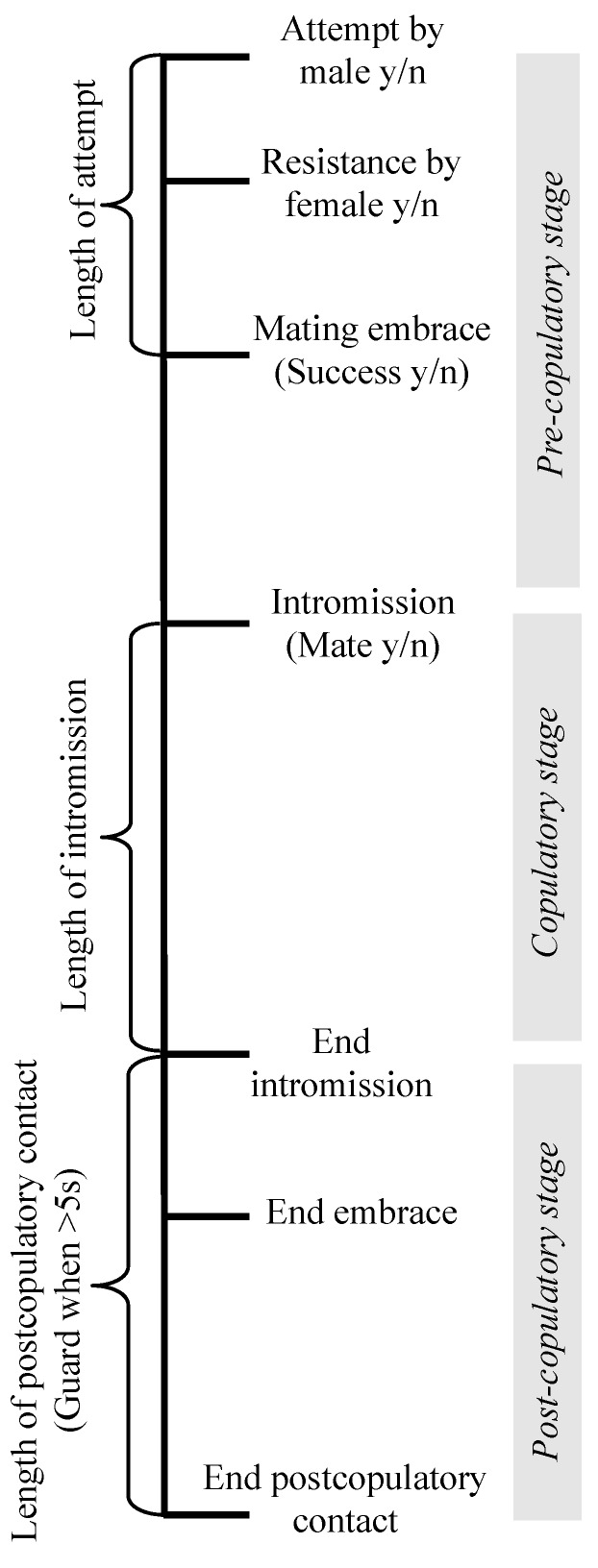
Timeline of behaviors during precopulatory, copulatory, and postcopulatory stages of mating interactions in leiobunine harvestmen. All of the behaviors measured are indicated in the order in which they occur on the timeline. The outcomes of each stage include: male attempt, yes or no (y/n); female resistance, yes or no; intromission (mating), yes or no; and postcopulatory guarding, yes or no.

Another aspect of mating in leiobunine harvestmen that makes them an attractive system for studying the evolution of mating behavior is that mating interactions follow a stereotyped series of stages in which different sources of selection appear to act [18]. Across the clade, mating occurs after a male secures a female in a mating embrace by hooking his pedipalps behind the coxae of her second legs (Figure 1A,B). There is a period of male–female interactions during which a male will deliver a nuptial gift, and then insert his genitalia into the female genital opening (Figure 1B). During intromission, females often tap or stroke the male genitalia, sometimes holding onto specialized structures on the distal portion of the hematadocha that inflates during the eversion of the male genitalia (e.g., in *Leiobunum vittatum*, Figure 1A) [18]. After intromission is complete, the pair disengages from the embrace. At this point, males may remain in contact with the females for a period of time, sometimes clasping or wrapping her legs to remain in contact [18] (Figure 1C). The timeline for mating interactions is illustrated in Figure 2.

We analyze whether male or female size predicts the success and timing of each stage of mating interactions in five species in the clade (Figure 1) that fall along the predicted antagonism spectrum. We aim to understand whether there are general patterns governing the role(s) of male and/or female size in predicting outcomes as mating interactions progress, and whether the role of size varies across species in accordance with variation in the level of predicted antagonism. We study two non-sacculate high-antagonism species: *Leiobunum vittatum* (for which mating behavior has already been studied in some detail [18]) and *L. calcar*. We contrast these with three sacculate species—*L. aldrichi*, *L. politum*, and *L. ventricosum*—with *L. aldrichi* and *L. politum* on the lower side of the antagonism spectrum, and *L. ventricosum* somewhere between low and high antagonism [24]. We predicted that the traits that predict mating outcome would vary across different stages of mating, because even in species with high antagonism, the interests of males and females should converge with the progression of mating as more time and energy is invested in the interaction [15,18]. We also predicted that species occupying similar parts of the antagonism continuum would show similar patterns in terms of which traits predicted the outcome of mating interactions. Our overall goal is to demonstrate the rich possibilities for understanding how selection shapes mating interactions in a greatly understudied group of organisms.

## 2. Materials and Methods

*Study organisms*—We collected the animals as mature individuals from four locations (year and GPS coordinates in parentheses): (i) *L. vittatum and L. aldrichi* from along the Milwaukee River in Milwaukee, Wisconsin (WI), United States (USA) (2013; 43.0428° N, 87.5331° W), (ii) *L. politum* from Mountain Lake Biological Station, Pembroke, Virginia (VA), USA (2014; 38.0342° N, 78.5129° W), (iii) *L. ventricosum* from the Katherine Ordway Natural History Study Area, Inver Grove Heights, Minnesota (MN), USA (2016; 44.8118° N, 93.0294° W), and (iv) *L. calcar* from Saint Louis, Missouri (MO), USA (2016; 38.5472° N, 90.5439° W). We identified species during collection using external morphological traits, and confirmed proper identification by examining species-specific genitalic traits [23,26]. Each individual was used in only a single mating trial. The mating history of individuals was unknown, but leiobunine harvestmen mate multiply (Fowler-Finn unpubl. [27]), and the individuals of all of the species have similarly unknown mating histories. However, we consider the potential for experience to confound results. 

We housed the animals in individual containers for one day to three weeks prior to using them in a mating trial. The containers were deli dishes (dimensions: 11-cm diameter × 8-cm depth) with holes in the lids and mosquito netting stretched across the top to allow for air flow and a substrate on which the animals could climb. The exception to this setup was *L. politum* at Mountain Lake Biological Station, for which the containers were 10 cm × 10 cm × 5 cm plastic containers, which is the best approximation available at the station. We fed all of the animals upon arrival in the lab, provided water *ad libitum*, and cleaned cages and freshened food (consisting of fish food and varied leftovers) and water twice weekly.

*Mating trials*—We conducted all of the mating trials in circular arenas (30-cm diameter) constructed from 22-cm high acetate walls and printer paper flooring [18]. To provide a backdrop for viewing videos, we surrounded the arena with white paper that was propped up ~10 cm from the arena walls (Figure 1). Between trials, we changed the paper on the floor, and wiped down the table and acetate paper with ethanol to remove potential chemical cues.

Prior to a trial, we gently placed individuals into the arena and placed a ~five cm diameter acetate barrier around the individuals for a two minute acclimation period (except in 2013, when males were allowed to roam the arena during acclimation, but females were contained). Trials started when we lifted the barriers and allowed individuals to freely interact, and ended when either mating was complete with no further attempts to remate, or when the female rejected the male three times (following Fowler-Finn et al. 2014 [18]). We video recorded all of the trials using handheld digital camcorders for later behavioral analyses.

Following Fowler-Finn et al. (2014) [18], we examined a standard set of behaviors in each trial: if an attempt occurred or not, if the attempt was successful or not (on the first try, as well as overall in the trial), if the female resisted the first attempt by a male, and if copulation occurred or not. Resistance included one of the following behaviors, defined in Fowler-Finn et al. (2014) [18]: the female runs away, vigorously bobs her body, bites the male, or orients her body in a head-down position to block male access to clasping her in the mating embrace. We also recorded if guarding occurred, which was counted when males stayed in contact with the female for five seconds or longer after the end of copulation. Finally, we also quantified the length of an attempt to secure the female in the mating embrace, the length of intromission, and the length of postcopulatory contact. See Fowler-Finn et al. (2014) [18] for detailed descriptions of the behaviors, and Figure 2 for a mating interaction timeline.

**Figure 3:**
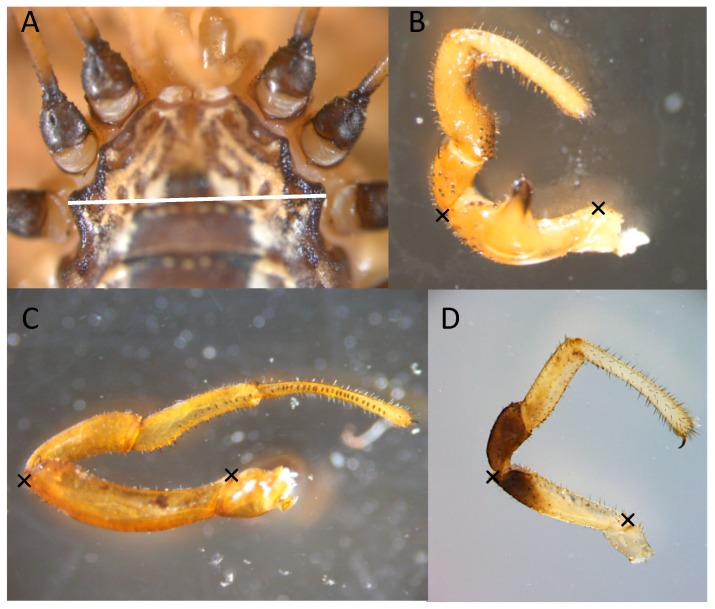
Morphological measurements taken of leiobunine harvestmen. (**A**) Cephalothorax width was measured at the widest point behind the second pair of legs (indicated by the white line). (**B**–**D**) Pedipalp length was measured as the distance on the femur between the indicated xs. The pedipalps pictured are (**B**) *L. calcar*, (**C**) *L. vittatum*, and (**D**) *L. aldrichi*.

*Morphological analyses*—We weighed individuals to the nearest 0.0001 g using a Mettler Toledo analytical balance at the end of each day. The timing of weighing allowed us to avoid disrupting the mating trials in the cases in which the animals released highly volatile alarm pheromones during weighing. All of the specimens were preserved in 70% ethanol at the conclusion of each experiment for morphological analyses. We measured the cephalothorax width at the widest point on the carapace between legs two and three as a measure of body size (Figure 3A). To do so, we oriented individuals in a standardized position under a Leica 205 C microscope fitted with a Leica MC170 HD microscope camera at 4× magnification (Saint Louis University, Saint Louis, MO, USA) or Olympus SZX10 microscope (*L. ventricosum* Macalester College, St Paul, MN, USA). Images were captured and later measured using Leica imaging software. We took two pictures per individual—removing and then reorienting the body in between photographs—and measured both images twice. The final cephalothorax width measurement was the mean of means of these photographs.

We measured male pedipalp femur length because males secure females in a mating embrace by hooking their pedipalps behind the female’s coxae of her second legs [18]. Furthermore, pedipalps have been shown to influence mating dynamics in *L. vittatum* [18], and also vary significantly across the antagonism spectrum, with larger pedipalps on the high antagonism end [23]. To do so, we first removed the right pedipalp (or the left when the right was damaged) and laid it in a stereotyped manner on a slide covered with a cover slip. We used the same microscope, camera, and image-processing software as we did for our body size measurements. Femur length was measured at the longest point-to-point distance diagonally across the pedipalp femur (Figure 3B–D). Similarly as to body size, we took two pictures per pedipalp, repositioning the pedipalp in between photos, and used the mean of means for the final measurement.

All of the researchers who took and processed images were trained in the same way by KD Fowler-Finn. Each researcher took two pictures and two measurements of each body part for 10 animals. Then, the repeatability of these measurements was verified with the variance component for individual identification (ID) in a mixed model analysis (*p* > 0.05, r > 0.98 for all datasets).

A single measure of ‘body size’ was calculated from the combination of weight and cephalothorax width. To do so, we ran a principal components analysis with weight and cephalothorax width, and retained the principal components with eigenvectors greater than 1.0. For each dataset, we identified only a single eigenvector explaining variation in body size for both males and females. We then used Pearson product moment correlations to determine if pedipalp length correlated with our measure of body size. Finally, we calculated sexual size dimorphism for each species for weight and cephalothorax width by dividing the male mean weight by female mean weight, and the male mean cephalothorax width by female mean cephalothorax width.

*Statistical analyses*—To determine the morphological predictors of each stage of mating for each dataset, we used nominal logistic regressions. Dependent variables included whether or not: the trial ended in mating (mate y/n), the male attempted (attempt y/n), the female resisted (resist y/n), the male secured the female on his first attempt (first attempt successful y/n), and the male eventually secured the female within three attempts (success y/n) (Figure 2). The dependent variable was whether the pair successfully moved to the next stage of the mating sequence or not. The independent variables were female size, male size, the interaction between female size and male size, and male pedipalpal femur length. To determine whether the morphological traits predicted the length of copulation and the length of postcopulatory contact, we used linear regressions with the same independent variables. 

Finally, following Fowler-Finn et al. (2014) [18], we looked at whether the relationship between the male–female size difference and attempt length differed depending on whether the attempt was successful or not. For all but *L. calcar* (which had a normal distribution), we log-transformed attempt length as the dependent variable. Independent variables were whether or not the attempt was successful, the size difference between females and males (female size–male size), and the interaction term between the size difference and whether the attempt was successful.

## 3. Results

### 3.1. Morphology

We found significant positive correlations between male body size and pedipalp length for *L. vittatum* and *L. aldrichi*, with marginally non-significant positive correlation in *L. politum* (Table 1). We found a range of sexual size dimorphisms across species, with *L. calcar* exhibiting the lowest dimorphism, and *L. ventricosum* exhibiting the largest dimorphism (Table 2).

### 3.2. Predictors of Outcome

*Predictors of overall outcome:* In three of the five species studied, we identified morphological predictors of whether or not mating occurred. For these species—*L. vittatum*, *L. calcar*, and *L. politum*—female size was the primary predictor (Table 3 and Table 4). For *L. aldrichi*, there was a marginally non-significant effect of female size on whether or not mating occurred. While larger females tended to mate more in *L. vittatum*, *L. politum*, and *L. aldrichi*, smaller females were more likely to mate in *L. calcar* (Table 3 and Table 4).

*Predictors of precopulatory interactions:* There were no clear patterns of morphological predictors for whether a male attempted to secure the female. In *L. calcar*, males were more likely to attempt to mate with smaller females, and in *L. aldrichi*, males were more likely to attempt to mate with larger females (Table 3 and Table 4). However, for *L. aldrichi,* these results are driven by a single male that did not attempt to mate with the smallest female in the experiment.

Whether or not the female resisted also had no clear patterns. In *L. vittatum*, resistance was more likely when interacting with males with shorter pedipalps; in *L. ventricosum*, resistance was more likely when the female was comparatively smaller than the male (Table 3 and Table 4). We did find that female body size was a primary predictor of whether a male was successful in securing a female, either in his first attempt or by three attempts (Table 3 and Table 4). Again, *L. calcar* showed the opposite pattern of the other species, with success being more likely with smaller females (Table 3 and Table 4).

The relationship between attempt length and the male–female size difference did not depend on whether the attempt was successful for any of the species tested (whole model: *p* > 0.05 for all of the species).

*Predictors of pericopulatory and postcopulatory outcomes:* The outcomes of pericopulatory and postcopulatory interactions were driven primarily by either male size, or an interaction between male and female size (Table 3 and Table 4). For the length of copulation: *L. vittatum* copulation lasted longer when the male was small, and in both *L. vittatum* and *L. ventricosum*, copulation was longer when the female was relatively larger than the male (Table 3 and Table 4). 

We found a mix of morphological predictors for the presence of guarding and the length of postcopulatory contact. In *L. vittatum*, males that were relatively smaller than the females were more likely to guard. In *L. politum*, larger males were more likely to guard, and also sustained postcopulatory contact for longer. Finally, in *L. ventricosum*, postcopulatory contact lasted longer when the female was larger.

## 4. Discussion

Male and female size predicted the outcomes and timing of various stages of mating in the five species of leiobunine harvestmen studied. However, these patterns varied across the mating stages as well as across species. We found that the success of precopulatory stages of mating was primarily predicted by female traits, whereas the success and length of copulatory and postcopulatory stages were primarily predicted by either male traits alone or the interaction between male and female traits. Despite this overall pattern, we found that the polarity of the relationship between size and outcome varied across species, and that this variation did not appear to correlate with the antagonism spectrum predicted by morphological characters in the clade [24]. 

The overall shift from female size predicting the outcome of early stages to male size predicting the outcome of later stages suggests some basic rules for mating interactions regardless of placement along the antagonism spectrum. The overall shift in predictors suggests that distinct sources of selection favoring different sets of traits may predominate during each stage of the mating process [18]. As male and female interests converge after they have assessed one another and approach fertilization [20], the sources of selection could shift [9,19], shaping different sets of traits [28]. For example, in earlier stages of mating interactions before males and females have had an opportunity to assess one another, tests of strength or size may dictate successful interactions, and later, more detailed assessments may occur [20], including an assessment of the traits favored by cryptic female choice such as nuptial gifts and sperm quantity [12]. Thus, it is not surprising that we observed a change in which traits predicted the outcome and timing of each stage of mating. 

During precopulatory stages of mating, larger females were more likely to mate and be successfully secured (except in *L. calcar*). Larger females could be more likely to mate because of higher receptivity due to being in better condition [29] or to being more gravid (i.e., containing more mature eggs); alternatively, males could show a preference for larger females [30,31,32,33]. We suggest an increased receptivity due to gravidity because we found no correspondence between female size and the likelihood of a male attempting to mate, suggesting little discrimination at that stage. Females are significantly larger than males (weighing ~50% more than males across most species), which may give them an advantage during the early stages of mating. 

In both *L. vittatum* and *L. ventricosum*, the length of intromission varied with male size and an interaction between male and female size, but the two species exhibited opposite patterns. Intromission was longer for smaller males in *L. vittatum*—which lack penile sacs—and longer for larger males in *L. ventricosum*—which possess penile sacs. Given the contrast in how size influences the length of this behavior, and the sacculate versus non-sacculate nature of the species, it seems reasonable to predict that different principles may guide intromission length in these two species. Long or repeated intromission can serve to increase sperm transmission [34,35], prolong contact for greater nuptial gift transfer—which could contribute direct benefits to females—or function as a means to gain access to mating or guard from future potential mates [36]. Also, when intromission time exceeds that necessary to transfer sperm, it can help reduce competition with other males [14,37,38] by reducing female receptivity or otherwise decreasing her chances of remating [13,14,39]. In leiobunine harvestmen, nuptial gifts are delivered prior to and during copulation, with preintromission delivery of the gift in sacculate species facilitated by sacs located just below the tip of the penis in some species [18,23,25,40]. For *L. vittatum*—a non-sacculate species—there is a prolonged period between the male securing the female and when intromission occurs; during the intervening time, males exhibit multiple insertions, and females appear to solicit nuptial gift delivery by males [18]. Assessing full intromission is difficult, and we counted the total time the penis was inserted as intromission, which encompasses both sperm and nuptial gift delivery. Smaller *L. vittatum* males could take longer to deliver a sufficient gift for the females to accept them as mates, or they could perceive their future potential mating success as low (i.e., low residual reproductive value), and be willing to invest more in current reproductive efforts [41,42,43]. 

For *L. politum*, males were more likely to guard females when the male was bigger. In contrast, for *L. vittatum* and *L. ventricosum,* the likelihood of guarding or length of postcopulatory contact was higher when females were larger. For males, prolonged guarding can lead to a loss of future mates [14,44,45], but it can also reduce sperm competition, potentially increasing male fitness depending on sperm precedence rules [14,44,46,47]. For females, guarding can allow for assessment and subsequent cryptic choice during postcopulatory contact [12]. We suspect the benefits of guarding are accrued through multiple mechanisms in the leiobunine clade. In *L. ventricosum,* mate-guarding may be a mechanism to increase repeated copulations, as males often remated with females after disengaging from the embrace; prolonged contact could therefore increase sperm transfer, which can increase fertilization success [48,49]. In *L. vittatum,* guarding could also exclude other male competitors; reduce the likelihood of female remating [45]; or, as suggested based on field observations, guarding can reduce female harassment during oviposition [50,51]. Interestingly, one species with very distinct postcopulatory behavior—*L. aldrichi*, in which males vigorously shake the female after mating (personal observation [52])—demonstrated no pattern of morphological correlates to postmating contact. Our ability to understand the patterns and mechanisms of sperm competition and fertilization, which are currently completely lacking in harvestmen, will help elucidate the potential sources of selection shaping the observed patterns of guarding and postcopulatory contact. However, the diversity of patterns observed in the current study suggests a clade that is rich with possibilities for studying the evolution of mate-guarding behavior.

The five species we studied have been previously classified to vary along an antagonism spectrum, with *L. aldrichi* and *L. politum* on the lower end, and *L. ventricosum*, *L. calcar,* and *L. vittatum* on the higher end [23,24]. The two species on the low end of the antagonism spectrum—*L. aldrichi* and *L. politum*—had the fewest morphological predictors of outcome. While this may be due to a lack of sample size in *L. politum,* we do note that *L. ventricosum* had fewer trials than *L. aldrichi*, but still had several predictors of outcome. In these sacculate species, females may rely more on the size and/or quality of nuptial gift to select mates. A new study on nuptial gift profiles has shown that the gifts of sacculate species contain a nearly 20% larger proportion of essential amino acids compared to non-sacculate species (Kahn et al., in press [53]), and this could be a significant trait that females assess in making mating decisions. 

We found significant differences in our dataset for *L. vittatum* compared with the 2014 study. Fowler-Finn et al. (2014) [18] found that successful attempts were shorter if females were larger, and unsuccessful attempts be longer when females are larger; this was interpreted as females making a decision and then resisting, which was a pattern we did not find in the current study. We also found other differences, notably including the higher success of males with shorter pedipalps in Fowler-Finn (2014). The current dataset encompasses a much wider range of collection dates, and we suggest the difference in results may reflect changes across a single mating season in mating dynamics, which we have found in multiple species in the clade (Fowler-Finn and Boyer, unpubl. data [54]). However, we also cannot fully rule out the potential for variation in experience in the field to shape some of the patterns that we describe for *L. vittatum* and the other species examined in the this study. The final interesting pattern that emerged from our study that is worth noting is that *L. calcar* differed from other species in that males were more successful across multiple stages of mating when females were smaller, and this species exhibited the lowest sexual dimorphism in weight among the species studied. 

## 5. Conclusions

We found that size predicted mating interaction outcomes in varied ways across species and stages of mating in leiobunine harvestmen, suggesting a rapid and complex evolution of mating behavior and assessment in this diverse clade. Overall patterns progress from primarily female size being the primary predictorof success in earlier stages of mating, to male size and male size relative to female size predicting success and duration of later stages. However, the polarity of size and its influence on mating outcomes varies dramatically across species, suggesting different mechanisms dictating the dynamics of various mating stages in different species. We also find contrasting patterns in size predictors across stages of mating, which suggest the action of multiple sources of selection, and suggest that mating success does not necessarily equate fertilization success. This study paints a picture of a clade that is rich for studying the evolution of mating behavior and decisions, and future work tackling relevant mechanisms is likely to reveal interesting results.

## Figures and Tables

**Table 1:** Correlations between the principal component describing male body size and pedipalp length generated using Pearson product-moment correlations. Bolded values indicate *p* < 0.05, and ‘n’ indicates the number of individuals used in the analysis. Species are arranged from low antagonism at the top of the table to high antagonism at the bottom, and categorized by the possession of penile sacs (sacculate) or the absence of penile sacs (non-sacculate).

Species	Correlation	*p*-Value	n
Sacculate			
*L. aldrichi*	**0.64**	**0.0011**	23
*L. politum*	0.53	0.0516	14
*L. ventricosum*	0.29	0.2031	21
Non-sacculate			
*L. calcar*	0.34	0.0624	31
*L. vittatum*	**0.53**	**0.0004**	41

**Table 2:** Variation in size (both cephalothorax width and weight) of male and female *Leiobunum* harvestmen of the five species studied. The mean ± S.E. are listed, as well as the sexual dimorphism for each size measurement per species, calculated from the male/female ratio. Species are arranged from low antagonism at the top of the table to high antagonism at the bottom of the table, and categorized by the possession of penile sacs (sacculate) or the absence of penile sacs (non-sacculate).

		Cephalothorax Width (mm)		Weight (g)		Cephalothorax Width Dimorphism	Weight Dimorphism
		Male	Female	Male	Female		
Sacculate							
*L. aldrichi*	mean	2.406	2.483	0.0212	0.0342	0.97	0.62
	S.E.	0.025	0.025	0.0006	0.0012		
*L. politum*	mean	2.788	2.946	0.0132	0.0270	0.95	0.49
	S.E.	0.0293	0.042	0.00054	0.0011		
*L. ventricosum*	mean	3.140	3.453	0.0485	0.1150	0.91	0.42
	S.E.	0.021	0.036	0.0001	0.0062		
Non-sacculate							
*L. calcar*	mean	3.839	3.838	0.1131	0.171	1.00	0.66
	S.E.	0.020	0.035	0.0023	0.0044		
*L. vittatum*	mean	2.966	3.061	0.0467	0.0854	0.97	0.55
	S.E.	0.016	0.015	0.0008	0.0016		

**Table 3:** Analyses testing for morphological predictors of various stages of mating in five species of leiobunum harvestmen. All of the models were either nominal logistic regressions or linear regressions. Bolded values indicated *p* < 0.05, and ‘n’ indicates the number of trials included in each analysis. The gray text in *L. aldrichi* indicates there was only one male that did not attempt. Sacculate species are on the left, and non-sacculate species are on the right, with the continuum of antagonism going from low antagonism to high antagonism, from left to right.

		Sacculate Species									Non-Sacculate Species					
		*L. aldrichi*			*L. politum*			*L. ventricosum*			*L. calcar*			*L. vittatum*		
		(7/23 Mated)			(11/14 Mated)			(18/21 Mated)			(22/31 Mated)			(20/41 Mated)		
		Χ^2^_(df)_	*p*	*n*	Χ^2^_(df)_	*p*	*n*	Χ^2^_(df)_	*p*	*n*	Χ^2^_(df)_	*p*	*n*	Χ^2^_(df)_	*p*	*n*
**Precopulatory:**																
Attempt y/n	female size	**7.6_(1,4)_**	**0.0060**	23	All attempted			0.0**_(1,4)_**	0.9960	21	**5.9_(1,4)_**	**0.0153**	31	0.1**_(1,4)_**	0.7686	41
	male size	0.0**_(1,4)_**	1.0000					0.0**_(1,4)_**	0.9988		0.0**_(1,4)_**	0.8592		0.8**_(1,4)_**	0.3865	
	female x male size	**4.0_(1,4)_**	**0.0451**					0.0**_(1,4)_**	0.9182		0.1**_(1,4)_**	0.7364		1.3**_(1,4)_**	0.2458	
	male pedipalp	0.0**_(1,4)_**	0.9983					0.0**_(1,4)_**	0.9991		0.3**_(1,4)_**	0.6069		1.5**_(1,4)_**	0.2252	
Resist y/n	female size	1.5**_(1,4)_**	0.2276	22	1.5**_(1,4)_**	0.2278	14	0.2**_(1,4)_**	0.6261	20	1.8**_(1,4)_**	0.1789	24	1.5**_(1,4)_**	0.2266	39
	male size	0.0**_(1,4)_**	0.8777		2.8**_(1,4)_**	0.0928		0.1**_(1,4)_**	0.7881		2.6**_(1,4)_**	0.1052		2.1**_(1,4)_**	0.1466	
	female x male size	0.0**_(1,4)_**	0.8402		2.3**_(1,4)_**	0.1283		**12.0_(1,4)_**	**0.0005**		1.7**_(1,4)_**	0.1911		1.2**_(1,4)_**	0.2655	
	male pedipalp	1.3**_(1,4)_**	0.2631		2.0**_(1,4)_**	0.1594		0.9**_(1,4)_**	0.3556		0.4**_(1,4)_**	0.5406		**8.5_(1,4)_**	**0.0036**	
First attempt successful	female size	1.9**_(1,4)_**	0.1647	22	1.5**_(1,4)_**	0.2200	14	0.7**_(1,4)_**	0.4129	20	**4.9_(1,4)_**	**0.0273**	24	**6.6_(1,4)_**	**0.0102**	39
	male size	0.0**_(1,4)_**	0.9732		**4.5_(1,4)_**	**0.0335**		1.0**_(1,4)_**	0.3096		1.1**_(1,4)_**	0.2947		0.9**_(1,4)_**	0.3332	
	female x male size	0.3**_(1,4)_**	0.5991		**4.4_(1,4)_**	**0.0350**		1.3**_(1,4)_**	0.2526		3.3**_(1,4)_**	0.0698		2.4**_(1,4)_**	0.1179	
	male pedipalp	0.3**_(1,4)_**	0.6107		**5.1_(1,4)_**	**0.0242**		0.3**_(1,4)_**	0.6055		0.3**_(1,4)_**	0.5544		0.3**_(1,4)_**	0.5714	
Secure y/n	female size	2.8**_(1,4)_**	0.0952	22	**4.1_(1,4)_**	**0.0432**	14	0.0**_(1,4)_**	0.9849	20	**4.9_(1,4)_**	**0.0273**	24	**9.9_(1,4)_**	**0.0017**	41
	male size	0.0**_(1,4)_**	0.9575		0.0**_(1,4)_**	0.9181		0.0**_(1,4)_**	0.9916		1.1**_(1,4)_**	0.2947		0.5**_(1,4)_**	0.4979	
	female x male size	0.4**_(1,4)_**	0.5413		0.0**_(1,4)_**	0.8359		80.1**_(1,4)_**	<0.0001		3.3**_(1,4)_**	0.0698		**4.4_(1,4)_**	**0.0361**	
	male pedipalp	0.2**_(1,4)_**	0.6785		1.7**_(1,4)_**	0.1910		23.8**_(1,4)_**	<0.0001		0.3**_(1,4)_**	0.5544		0.3**_(1,4)_**	0.5837	
**Copulatory:**																
Mate y/n	female size	3.4**_(1,4)_**	0.0669	23	**4.1_(1,4)_**	**0.0432**	14	0.1**_(1,4)_**	0.7477	21	**11.7_(1,4)_**	**0.0006**	31	**6.1_(1,4)_**	**0.0136**	41
	male size	0.0**_(1,4)_**	0.9806		0.0**_(1,4)_**	0.9181		2.3**_(1,4)_**	0.1292		0.3**_(1,4)_**	0.5567		0_(1,4)_	0.8329	
	female x male size	0.4**_(1,4)_**	0.5508		0.0**_(1,4)_**	0.8359		2.1**_(1,4)_**	0.1464		2.1**_(1,4)_**	0.1487		1.3_(1,4)_	0.2570	
	male pedipalp	0.2**_(1,4)_**	0.6765		1.7**_(1,4)_**	0.1910		0.7**_(1,4)_**	0.3998		1.1**_(1,4)_**	0.2995		0.6_(1,4)_	0.4481	
Length Intromission	fem size	0.1**_(1,4)_**	0.8352	6	0.0**_(1,4)_**	0.8484	11	1.8**_(1,4)_**	0.2047	18	0.0**_(1,4)_**	0.9347	22	1.8**_(1,4)_**	0.2048	20
	male size	0.0**_(1,4)_**	0.9780		0.0**_(1,4)_**	0.9965		4.5**_(1,4)_**	0.0534		3.4**_(1,4)_**	0.0837		**11.7_(1,4)_**	**0.0038**	
	female x male size	0.1**_(1,4)_**	0.7946		0.0**_(1,4)_**	0.9214		**7.1_(1,4)_**	**0.0194**		0.8**_(1,4)_**	0.3912		**7.2_(1,4)_**	**0.0170**	
	male pedipalp	0.0**_(1,4)_**	0.9629		0.7**_(1,4)_**	0.4219		0.7**_(1,4)_**	0.4070		0.5**_(1,4)_**	0.4857		0.2**_(1,4)_**	0.7030	
**Postcopulatory:**																
Guard y/n	female size	All guarded		6	0.0**_(1,4)_**	0.9083	11	2.4**_(1,4)_**	0.1221	18	0.1**_(1,4)_**	0.7790	21	3.7**_(1,4)_**	0.0542	20
	male size				**4.8_(1,4)_**	**0.0282**		2.3**_(1,4)_**	0.1295		2.8**_(1,4)_**	0.0971		0.5**_(1,4)_**	0.4717	
	female x male size				0.2**_(1,4)_**	0.6770		0.7**_(1,4)_**	0.4064		0.5**_(1,4)_**	0.4898		**4.5_(1,4)_**	**0.0332**	
	male pedipalp				0.0**_(1,4)_**	0.9083		1.1**_(1,4)_**	0.2865		1.7**_(1,4)_**	0.1986		0.6**_(1,4)_**	0.4492	
Length postcopulatory contact	female size	58.9_(1,4)_	0.0825	6	0.9**_(1,4)_**	0.3887	11	**7.0_(1,4)_**	**0.0200**	**18**	0.6**_(1,4)_**	0.4661	21	1.3**_(1,4)_**	0.2746	20
	male size	33.4_(1,4)_	0.1090		**7.0_(1,4)_**	**0.0384**		3.0**_(1,4)_**	0.1053		0.5**_(1,4)_**	0.4961		0.0**_(1,4)_**	0.9463	
	female x male size	35.7_(1,4)_	0.1055		3.1**_(1,4)_**	0.1288		1.6**_(1,4)_**	0.2235		0.3**_(1,4)_**	0.5941		2.2_(1,4)_	0.1602	
	male pedipalp	28.8_(1,4)_	0.1172		3.5**_(1,4)_**	0.1115		4.2_(1,4)_	0.0600		1.5**_(1,4)_**	0.2427		0.8**_(1,4)_**	0.3935	

**Table 4:** How the size of males and females predict the success and timing of each stage of mating in the five species of leiobunine harvestmen studied. The size of the male and female symbol indicate whether larger or smaller males/females were more likely to either be successful at a given stage, or more likely to have a longer duration of a given stage. When the relative size of males versus females predicted outcome, the ‘<’ and ‘>’ symbols indicate the relationship dictating the most successful or longest duration of a stage. The “—” indicates that neither male nor female body size predicted outcome. Sacculate species are on the left, and non-sacculate species are on the right with the continuum of antagonism going from low antagonism to high antagonism from left to right.

		Sacculate			Non-Sacculate	
		*L. aldrichi*	*L. politum*	*L. ventricosum*	*L. calcar*	*L. vittatum*
Precopulatory	Attempt y/n	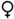 (One did not attempt)	—	—	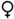	—
	Resist y/n	—	—	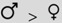	—	—
	First attempt successful	—	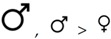	—	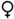	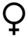
	Secure y/n	—	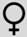	—	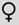	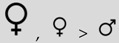
Copulatory	Mate y/n	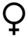	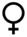	—	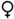	
	Length intromission	—	—	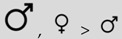	—	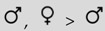
Postcopulatory	Guard y/n	—	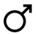	—	—	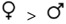
	Postcopulatory contact	—	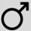	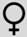	—	—
